# Zoobiquity: What Animals Can Teach Us About Health and the Science of Healing

**DOI:** 10.3390/ani2040559

**Published:** 2012-10-01

**Authors:** Ray Greek

**Affiliations:** Americans for Medical Advancement, 2251 Refugio Rd., Goleta, CA 93117, USA; E-Mail: DrRayGreek@gmail.com; Tel.: +1-805-685-6812.

*Zoobiquity: What Animals Can Teach Us About Health and the Science of Healing* (Knopf 2012) is an easy to read and entertaining book co-written by Barbara Natterson-Horowitz, MD and Kathryn Bowers. Natterson-Horowitz is a practicing cardiologist at the University of California, Los Angeles (UCLA) David Geffen School of Medicine, who also has training in psychiatry. Kathryn Bowers is a professional writer who teaches writing at UCLA. The book addresses traits shared by nonhuman animals (hereafter referred to simply as animals) and humans that have medical relevance. The authors are to be commended for discussing matters that should be obvious in the 21st century, but sadly still are not universally accepted. Humans share our lineage with animals and this has implications for the origin of traits. Clearly, animals have emotions, preferences, and suffer from diseases that are similar on some levels to the ones humans suffer from. The Cartesian view of animals has been debunked and the authors give many examples supporting a more scientifically advanced view of animals. 

The theme of *Zoobiquity *is more far reacing, however. The authors contend that because animals and humans share an evolutionary history, we should expect them to also share diseases and that physicians and veterinarians could learn more about these diseases, and hence how to treat their patients, by better communicating with each other and being aware of the influence evolutionary history has on diseases. While this theme has merits and is right in what it confirms, it is unfortunately wrong in what it denies.

I am a physician and my wife, Jean Greek, is a veterinarian. As we were completing residency training in anesthesiology (RG) and dermatology (JG), we had very similar thoughts to those expressed in *Zoobiquity*. We recognized both similarities and differences among animals and humans and wondered if more communication might benefit both physicians and veterinarians. However, as we pursued this notion, we began to learn that the very small differences among species outweighed, in large part, the similarities in terms of medical intervention. We also eventually concluded that there is a difference in quality of care between vets and physicians but that this revolves around the money available for treating humans (more) as opposed to animals (less), not the intelligence or dedication of the practitioner. 

Along the way, we encountered Evolutionary Medicine, also called Darwinian Medicine, the area of science, which *Zoobiquity* ostensibly addresses. Evolutionary Medicine challenges the medical community to place diseases in the context of evolution. When this is carried out, some the interventions commonly performed in medicine might need to be abandoned. For example, do we really need to prescribe anti-pyretics when a patient has a fever with no other symptoms or signs? Evolutionary Medicine also addresses topics like *why* humans suffer from the diseases we do, along with what can be learned and implemented from this knowledge. In short, studying evolution can be a valuable heuristic (an aid in learning) for discovering more about diseases. We embraced this concept.

If this were the theme of *Zoobiquity*, it would be an interesting book is written in the form of a series of entertaining stories about how animals and humans manifest what are arguably versions of the same diseases. The theme of *Zoobiquity* is far more than *comparative medicine can be used as a heuristic*, however. *Zoobiquity* begins by quoting the 19th century, German physician and pathologist Rudolf Virchow: “between animal and human medicine there are no dividing lines—nor should there be. The object is different but the experience obtained constitutes the basis of all medicine.” The authors go on to discuss the Canadian physician Sir William Osler who studied under Virchow and agreed with him on the “no dividing lines” notion. This concept is currently popular in the form of *One Health*, defined as: “the collaborative effort of multiple disciplines—working locally, nationally, and globally—to attain optimal health for people, animals and the environment” [1]. This concept is echoed by various universities such the University of Pennsylvania: “Many Species. One Medicine.™—The concept of one single medicine for all species” [2]. And this is where the problem lies. 

Woven in among all the verbiage describing the concern for animal health, “One Health” and “Many Species. One Medicine”™ purport that research using animals, regardless of how it is carried out, in the course of diagnosing and treating pets, or studying animals in the wild, or studying animals in laboratories, is directly translatable to humans and vice-versa. Natterson-Horowitz and Bowers confirm this, with an emphasis on animals in the wild, in saying: “We could improve the health of all species by learning how animals live, die, get sick and heal in their *natural* settings. . . . I wanted to break down the wall between physicians, veterinarians, and evolutionary biologists because together we are uniquely situated to explore the animal-human overlap where it matters most urgently—in the effort to heal our patients” (p10, 16) If this were not clear enough, psychiatrist and Darwinian Medicine advocate Randolph Nesse stated: “What Barbara is doing is very important. Not just to break down barriers between disciplines and to help us realize we are yet another species shaped by selection, but also because insights from diseases in other animals will be helpful in understanding, preventing and treating human diseases” [3]. Evolutionary medicine is an important and neglected area of study but the reasoning manifest in *Zoobiquity*, along with claims of direct applicability among species, do nothing to further its acceptance. 

The specific examples used by the authors are reflective of the problems with the concept of zoobiquity. Psychogenic alopecia is an antiquated diagnosis as such self-induced hair loss is nearly always secondary to allergies and resolves when pruritus is addressed. Allergy is an unlikely etiologic factor in human self-mutilators. Equine melanoma in gray horses is cited as a corollary to the disease in humans. However, the disease in horses is not sun-induced nor is it particularly aggressive. My own horse has had a melanoma underneath her tail, which has not progressed for a dozen years. Humans with that diagnosis would be unlikely to be so fortunate. The authors go on to conflate a totally unrelated sun induced disease, which occurs on the “white socks” of horses legs, reporting that many horse owners apply “zinc oxide” to these areas. Unfortunately for the notion of zoobiquity, the disease that these horse owners are attempting to prevent is a photo-activated vasculitis that occurs after the ingestion of certain weeds. While all the factors that induce melanoma in humans have no doubt not been ascertained, I will certainly be surprised to learn that the disease is caused by eating weeds. 

These examples notwithstanding, the reason animals can be used as a heuristic but not to directly extrapolate results to humans [4,5,6,7] lies in a deeper understanding of *evolved complex systems*. Humans and animals are complex, as opposed to simple, systems and as such exhibit the properties of complex systems. These properties include the following.

1. Complex systems are very dependent on initial conditions, such as the genetic makeup of the individual. Dependence on initial conditions can be illustrated by the fact that monozygotic twins, because of very small differences in genetic makeup, can differ in their susceptibility to diseases such as schizophrenia. Along the same lines, different strains of mice vary dramatically in response to knocking out the same gene.
2. Complex systems display a hierarchy of levels of organization where different levels can react differently to the same perturbation.
3. Complex systems respond to perturbation such as drugs and disease in a nonlinear fashion. Seemingly small perturbations may lead to death while larger ones may not impact the system at all.
4. Complex systems have emergent properties that cannot be determined by reductionism. Complex systems are more than the sum of their parts, which again results in reductionism alone being insufficient to understand the intact complex system. A complex system must be studied as a whole and this limits what can be learned about one complex system by studying another complex system, regardless of how similar the two are.

Humans are also the product of evolution and the process has resulted in species having very different initial conditions due to mutations, differences in how the same genes are regulated and expressed, convergent evolution where a trait is manifest in two different lineages but the mechanism of the trait varies between the species, and a host of other factors. The eye of humans and octopi is an example of the same trait being acquired by very different mechanisms. The wiring of the human eye differs dramatically from the wiring of the cephalopod eye thus limiting what can be learned about one from studying the other. Efficacy and safety of treatments, even when species share the same physiology or mechanism of disease, may also vary. 

Differences in gene regulation alone have profound implications. Examples include the fact that the caterpillar and butterfly both have the same genes, but regulate and express them differently resulting very different appearing organisms. Another example is the fact that mice and humans share the gene that grows a tail in mice. The gene is not turned on during human development; hence humans do not normally manifest a tail. Think of genes as the keys on a piano and the regulatory genes as the sheet music. All pianos share the same keys but, depending on the sheet music, one can produce a wide variety of different tunes by activating the shared keys in various sequences. The recent ENCODE project [8] illustrates the amount of DNA involved in regulation. The percentage of genes shared between species is thus irrelevant solely in light of differences in regulation and expression. More or less all mammals could be constructed from the same genetic toolkit simply by regulating and expressing the same genes differently during embryogenesis. 

Place these evolution-derived differences in the context of a complex system and the stage is set for completely different outcomes to the same perturbation to the system. This is in fact what is observed in response to perturbations such as drugs and disease. Combine this lack of depth regarding evolutionary biology with the fact that the authors seem to assume that everything is an adaptation and the scientific foundation for *Zoobiquity* is mortally compromised. Further add to this the fact that even humans vary dramatically in their response to drugs and disease, leading to the concept and field known as *personalized medicine*, and one can hang a “Do Not Resuscitate” order on One Health and zoobiquity.

I found the attitude of the authors to be Pollyannaish, reminiscent of my own history, and wondered what other reviewers had written. To my surprise, almost every review was complimentary. The one exception that I found was from a veterinarian specializing in the care of animals in laboratories. She pointed out, correctly, that the concepts discussed in *Zoobiquity* have been appreciated and practiced for years by researchers and veterinarians who study animal models of disease. There is even a class of animal models referred to as *spontaneous*, meaning the study of animals that spontaneously suffer from the disease as opposed to *induced* models, meaning the disease must be artificially created in the animal. Indeed, the logical extension of *Zoobiquity* and One Health is the laboratory, where animals have been studied for decades but where there exists a profound lack of translation between species [5,6,7,9]. In short, while zoobiquity has been great in terms of comparative studies, it has been tried and has failed in terms of predicting human response to drugs and disease.

The authors present myriad facts but most of these do not directly relate to the case they are trying to make. The interesting and true parts of *Zoobiquity* are simply comparative biology and are not ground breaking nor are they vital for medical advancement. The important aspects of response to drugs and disease require a deeper examination. Yes, animals and humans share traits such as the susceptibility to be infected by viruses and the presence of hearts that are susceptible to diseases. But these are merely surface commonalities or traits manifest on the gross level of examination and hence do not imply the same mechanisms, natural history, or etiology of a disease. Deeper examination reveals that the mechanism by which a virus such as HIV infects the cell differs dramatically among species, as does the resulting illness. “A zoobiquitous approach to cancer,” advocated by the authors, has been the focus of the War on Cancer and has failed because species differ in cancer-causing mechanisms and this can be explained by the fact that animals and humans are evolved complex systems that are differently complex. Animal models of cancer, be they induced or spontaneous have not resulted in: “how oncologists might search for ways to cure it [cancer]” (p12). The fact that other species suffer from cancer is so well known as to be considered trivial in a book purporting to break new ground in the search for cures. 

In the final analysis, the stories the authors relate, while interesting, are examples of what are called “just so stories,” after a collection of stories by the same name by Rudyard Kipling. In the stories, Kipling explains things like how leopards got their spots as well as other interesting traits seen in animals. Speculation regarding the origin of traits in animals, put forward without supporting evidence, is the basis of the just so story and is not taken seriously by scholars as they are a form of the *ad hoc* fallacy. While just so stories may someday gain supporting evidence, the fact that they have none when proposed is what distinguishes them from science. Just so stories may eventually contribute to our understanding of the material universe, but they have no place in a book posing as a serious scholarly work and that sets as its purpose: the finding of cures.

I found no mendacity in *Zoobiquity*, but sadly it also lacks 21st century science. 
